# Supplementary material for: Complex‐centric proteome profiling by SEC‐SWATH‐MS
Source: Mol Syst Biol. 2019 Jan 14;15(1):e8438. doi: 10.15252/msb.20188438 (PMC6346213; doi:10.15252/msb.20188438)
Supplement: Supplementary file 8 — Dataset EV7 [file MSB-15-e8438-s008.zip › feature_plots_string/Q13242.pdf]

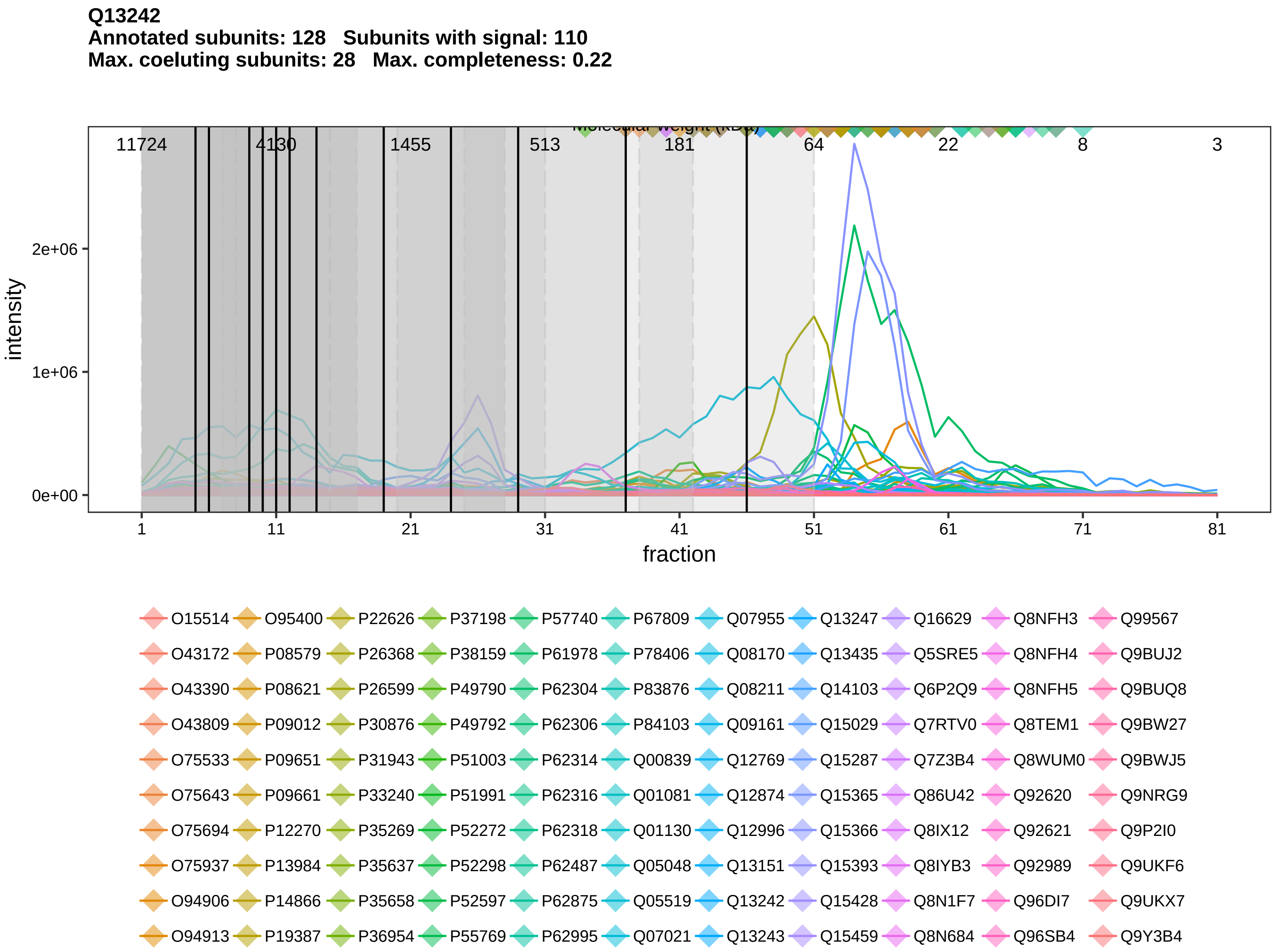

Q13242
Annotated subunits: 128 Subunits with signal: 110
Max. coeluting subunits: 28 Max. completeness: 0.22
11724
4130
1455
513
181
64
22
8
3
intensity
2e+06
1e+06
0e+00
1
11
21
31
41
51
61
71
81
fraction
O15514
O43172
O43390
O43809
O75533
O75643
O75694
O75937
O94906
O94913
O95400
P08579
P08621
P09012
P09651
P09661
P12270
P13984
P14866
P19387
P22626
P26368
P26599
P30876
P31943
P33240
P35269
P35637
P35658
P36954
P37198
P38159
P49790
P49792
P51003
P51991
P52272
P52298
P52597
P55769
P57740
P61978
P62304
P62306
P62314
P62316
P62318
P62487
P62875
P62995
P67809
P78406
P83876
P84103
Q00839
Q01081
Q01130
Q05048
Q05519
Q07021
Q07955
Q08170
Q08211
Q09161
Q12769
Q12874
Q12996
Q13151
Q13242
Q13243
Q13247
Q13435
Q14103
Q15029
Q15287
Q15365
Q15366
Q15393
Q15428
Q15459
Q16629
Q5SRE5
Q6P2Q9
Q7RTV0
Q7Z3B4
Q86U42
Q8IX12
Q8IYB3
Q8N1F7
Q8N684
Q8NFH3
Q8NFH4
Q8NFH5
Q8TEM1
Q8WUM0
Q92620
Q92621
Q92989
Q96DI7
Q96SB4
Q99567
Q9BUJ2
Q9BUQ8
Q9BW27
Q9BWJ5
Q9NRG9
Q9P2I0
Q9UKF6
Q9UKX7
Q9Y3B4
